# Mediterranean Dietary Pyramid

**DOI:** 10.3390/ijerph18094568

**Published:** 2021-04-26

**Authors:** Walter Willett

**Affiliations:** Departments of Nutrition and Epidemiology, Harvard T.H. Chan School of Public Health, Boston, MA 02115, USA; wwillett@hsph.harvard.edu

The updated Mediterranean Dietary Pyramid (MDP) described by Serra-Majem et al. [1] is a highly welcome addition to the nutritional landscape, built on the solid foundations of earlier versions. As its construction over the last decade was a collective effort by an expanded circle of experts representing additional countries around the Mediterranean region, this update incorporates additional insights on regional dietary traditions and engages a broader network in the transformation of our food systems to be healthier and more sustainable. Although many new scientists have contributed to this effort, I am pleased to note the input of Antonia Trichopoulou, who has been a consistent, knowledgeable thread connecting the first MDP with this update. 

The description of the foods comprising this updated pyramid has been fine tuned to be more specific and emphasize the central role of healthy plant foods and the modest role of meat, especially red meat, and dairy. This is consistent with Mediterranean dietary traditions, and the evidence linking this pattern with better health has strengthened with time. Notably, the EAT-Lancet commission, charged with identifying a pathway to healthy and sustainable diets for the expected world population by 2050, reviewed the available evidence on diet and health to develop global targets for specific food groups. When these targets were combined into an overall dietary pattern, they aligned tightly with the traditional Mediterranean diet as described in the updated MDP. This was invaluable proof that these targets were not just a conceptual goal but were consistent with a way of eating that had been tested over millennia and were found to be associated with well-documented longevity and wellbeing. 

The fundamental innovation in the updated MDP is a dimension representing environmental sustainability. When my colleagues and I designed the first Traditional MDP in 1993, health effects were our focus. This was impactful because the emphasis of dietary guidelines had been to reduce dietary fat as much as possible and encourage high carbohydrate intake. This was not consistent with scientific evidence emerging at that time, and the MDP made a transformative statement that the type of fat, not total fat, was important for long-term health. At that time, we appreciated the importance of the environmental impacts of food systems, but most of us thought that the impact of climate change would be felt centuries into the future. Since then, global warming has accelerated, and we are already experiencing adverse effects in many ways. At present, we are far off track to achieve the United Nations goal of limiting temperature increases by 2100 to less than 2 degrees C; instead, we are on course for disastrous and irreversible environmental changes. Thus, environmental sustainability has become an urgent issue and the addition of this new dimension in the updated MDP is important and timely. Notably, work cited in this report suggests that the adoption of the traditional Mediterranean dietary pattern could reduce greenhouse gas emissions from the food system by half or more compared to current Western diets. This, combined with transition to green energy sources, could enable us to pass on a sustainable world to future generations. All countries can benefit by considering this updated MDP when developing their dietary guidelines and food systems. 

## Data Availability

No new data were created or analyzed in this study. Data sharing is not applicable to this article.
